# Conformal Fabrication of an Electrospun Nanofiber Mat on a 3D Ear Cartilage-Shaped Hydrogel Collector Based on Hydrogel-Assisted Electrospinning

**DOI:** 10.1186/s11671-021-03571-6

**Published:** 2021-07-09

**Authors:** Jin Yeong Song, Hyun Il Ryu, Jeong Myeong Lee, Seong Hwan Bae, Jae Woo Lee, Changryul Claud Yi, Sang Min Park

**Affiliations:** 1 School of Mechanical Engineering, Pusan National University, 2, Busandaehak-ro 63 beon-gil, Geumjeong-gu, Busan, 46241 South Korea; 2 Department of Plastic and Reconstructive Surgery, Pusan National University School of Medicine, 179 Gudeok-ro, Seo-gu, Busan, 49241 South Korea; 3 Biomedical Research Institute, Pusan National University Hospital, 179 Gudeok-ro, Seo-gu, Busan, 49241 South Korea

**Keywords:** Electrospinning, Nanofiber, Hydrogel, 3D printing, Ear cartilage

## Abstract

Electrospinning is a common and versatile process to produce nanofibers and deposit them on a collector as a two-dimensional nanofiber mat or a three-dimensional (3D) macroscopic arrangement. However, 3D electroconductive collectors with complex geometries, including protruded, curved, and recessed regions, generally caused hampering of a conformal deposition and incomplete covering of electrospun nanofibers. In this study, we suggested a conformal fabrication of an electrospun nanofiber mat on a 3D ear cartilage-shaped hydrogel collector based on hydrogel-assisted electrospinning. To relieve the influence of the complex geometries, we flattened the protruded parts of the 3D ear cartilage-shaped hydrogel collector by exploiting the flexibility of the hydrogel. We found that the suggested fabrication technique could significantly decrease an unevenly focused electric field, caused by the complex geometries of the 3D collector, by alleviating the standard deviation by more than 70% through numerical simulation. Furthermore, it was experimentally confirmed that an electrospun nanofiber mat conformally covered the flattened hydrogel collector with a uniform thickness, which was not achieved with the original hydrogel collector. Given that this study established the conformal electrospinning technique on 3D electroconductive collectors, it will contribute to various studies related to electrospinning, including tissue engineering, drug/cell delivery, environmental filter, and clothing.

**Supplementary Information:**

The online version contains supplementary material available at 10.1186/s11671-021-03571-6.

## Introduction

Electrospinning is an effective and versatile technique for fabricating nanofibers and their assemblies, which has been extensively studied over the past decades [1]. Due to their unique properties such as high porosity, high surface-to-volume ratio, and extracellular matrix-mimicking structure [2], extraordinarily electromagnetism, electrospun nanofibers, and their assemblies have been created substantial interests from various research fields, including clothing [3], environmental filter [4–6], battery [7], and tissue-engineered scaffolds [8–10]. However, the chaotic motion of electrospun nanofibers due to bending instability has hampered the accurate and precise control in constructing nanofiber assemblies and generally created randomly interwoven 2D nanofiber mat [11]. Recent advances in the electrospinning process have expanded the range of the nanofiber assemblies from a 2D flat nanofiber mat to a 3D nanofibrous macrostructure [12–15]. With 3D nanofibrous scaffold fabricating techniques, including 3D template electrospinning, nanofiber film stacking, and centrifugal spinning [13–16], various 3D nanofibrous macrostructures have been fabricated, including a hollow tubular shape [17], molded 3D complex geometries [18], and native tissue-shaped nanofibrous scaffolds [19]. Such 3D nanofibrous macrostructures have become a growing topic of interest, particularly in tissue engineering, due to not only possessing a biomimetic nanofibrous structure but also mimicking a 3D macrostructure of native tissues [20, 21]. As a result, various researches have led to an improvement in the aesthetic and performance functions of 3D nanofiber assemblies [22, 23].

Among various 3D electrospinning techniques, including hydrolysis, gas-forming techniques, rolling, folding, and 3D electroconductive collector, electrospinning on a 3D electroconductive collector provided a facile way to assembly electrospun nanofibers into a 3D nanofibrous macrostructure without any post-processing [24–27]. The complex geometries of the 3D electroconductive collector, such as a large bending or a recessed shape, enabled to produce aligned nanofiber mats or fluffy 3D nanofibrous macrostructures [28]. However, such complex geometries, at the same time, hampered the deposition of electrospun nanofibers on the recessed region of the collector and caused incomplete covering of the 3D electroconductive collector. Thus, it has been greatly difficult to replicate the complex geometries of the 3D electroconductive collector to a 3D nanofiber mat. Considering that such incomplete covering could cause degradation of functionalities such as filtering efficiency and mechanical properties, it is necessary to develop a technique that replicates the shape of a 3D electroconductive collector with high complexity into a 3D nanofibrous macrostructure to broaden the application of electrospun nanofiber assemblies.

In this study, we proposed a conformal fabrication of an electrospun nanofiber mat that replicates the shape of a 3D electroconductive collector with complex geometries. As a representative example of the 3D electroconductive collector, we selected a 3D ear cartilage-shaped collector for the conformal fabrication of an electrospun nanofiber mat due to its complex geometries. In the previous study, we suggested a hydrogel, which possessed sufficient mobile ions to generate the electric field like a metal collector, as an electroconductive collector for electrospinning [29]. Here, we utilized the flexibility of a hydrogel collector to conformally and uniformly deposit electrospun nanofibers on the 3D ear cartilage-shaped hydrogel collector. Unlike a metal collector, the hydrogel collector could be flattened due to the flexibility of the hydrogel and, thereby, generate a uniform electric field to evenly deposit nanofibers on an entire surface of the 3D ear cartilage-shaped hydrogel collector. Furthermore, the mechanical strength of the hydrogel could be adjusted to that of the native tissue by finding an appropriate hydrogel. We selected alginate and gelatin as hydrogel materials due to their excellent biocompatibility [30, 31]. By mixing alginate and gelatin, we could attain mechanical properties similar to the native ear cartilage [32]. We numerically investigated the influence of the flattening of the 3D ear cartilage-shaped hydrogel collector on the conformal fabrication of an electrospun nanofiber mat on the collector. Finally, we confirmed the conformal fabrication of an electrospun nanofiber mat on the 3D ear cartilage-shaped hydrogel collector by measuring the thickness of the electrospun nanofiber mat at several positions, including helix, antihelix, scapha, and antitragus.

## Materials and Methods

### Materials

Sylgard® 184 silicone elastomer base of polydimethylsiloxane (PDMS) monomer and Sylgard® 184 silicone elastomer hardener of the curing agent were purchased from Dow Corning (USA). Polylactic acid (PLA) filaments were obtained from Snapmaker (USA). Gelatin from bovine skin, sodium salt of alginate acid, calcium chloride dihydrate (≥ 99%), polycaprolactone (PCL, Mw 80000), and chloroform (≥ 99.5%) were prepared from Sigma Aldrich (USA). Deionized water and methyl alcohol (≥ 99.5%) were obtained from Samchun Chemical Co., Ltd. (South Korea). All materials were used without additional purification.

### Fabrication of a PDMS Negative Mold of the 3D Ear Cartilage-Shaped Template

The CAD file of the 3D ear cartilage-shaped template with a dimension of 70 (length) × 20 (width) × 1.1 mm (thickness) was obtained through the Turbosquid website and modified in 3DS Max. The 3D ear cartilage-shaped template was then printed by a 3D printer (A150, Snapmaker, USA). PDMS monomer and curing agent were mixed at a ratio of 10:1. The uncured PDMS mixture was stirred manually for 5 min for uniform mixing and then degassed in a vacuum chamber until all visible air bubbles disappeared. When all air bubbles disappeared, the uncured PDMS mixture was poured into a disposable weighing dish, and the 3D ear cartilage-shaped template in the dish was utterly immersed in the PDMS mixture. The dish was placed into the oven and cured at 50 °C for 24 h. After curing, the dish was cut in half, and the 3D ear cartilage-shaped template was removed to obtain a PDMS negative mold.

### Preparation of an Alginate–Gelatin Hydrogel

Four alginate–gelatin hydrogels were prepared with different weight ratios (Table 1). Gelatin was dissolved in 50 °C water by a magnetic stir at 300 rpm for 1 h. Alginate was then added and mixed manually for 5 min. Then, an alginate–gelatin gel solution was poured into the PDMS negative mold. The alginate–gelatin gel solution was ionically crosslinked for 2 h in 10% w/w calcium chloride solution. After that, an alginate–gelatin hydrogel was departed from the PDMS negative mold and utilized as an electroconductive collector for electrospinning.

**Table 1:** Mixing components of the hydrogel sample

Hydrogel sample	Alginate	Gelatin	Deionized water
A0G100	–	3 g	15 g
A100G0	3 g	–	15 g
A25G75	0.75 g	2.25 g	15 g
A50G50	1.5 g	1.5 g	15 g

A0G100, gelatin; A100G0, alginate; A25G75, alginate/gelatin = 25:75; A50G50, alginate/gelatin = 50:50

### Mechanical Test of an Alginate–Gelatin Hydrogel

The alginate–gelatin hydrogels were prepared with the shape of an ASTM D638 Type IV specimen to measure mechanical properties by the tensile test. Each prepared hydrogel specimen was loaded on a universal test machine (QM100S, QMESYS, South Korea). The tensile test was conducted under a constant displacement at a speed of 10 mm min^−1^. The elastic modulus and ultimate tensile strength (UTS) of the specimen were calculated from the stress–strain curve.

### Conformal Fabrication of an Electrospun Nanofiber Mat

PCL (7.5%, w/v) was dissolved in chloroform–methanol (3:1) with stirring over 6 h. A PCL solution was placed in a 3-mL plastic syringe, and a syringe pump (NE-1000, New Era Pump Systems, Inc., USA) ejected the PCL solution through the metal needle with a flow rate of 0.4 mL h^−1^. The 3D ear cartilage-shaped hydrogel collector of the alginate–gelatin hydrogel with ratio of 25:75, namely A25G75, was placed on a polymethyl methacrylate (PMMA) flat substrate, and the PMMA substrate was located 20 cm below the metal needle. For electrospinning, a high voltage of 19 kV (HV30, NanoNC Co., Ltd., South Korea) was applied between a 23-gauge metal needle with an inner diameter of 0.6 mm and the 3D ear cartilage-shaped hydrogel collector under room temperature and controlled humidity of about 40–50%. To conformally deposit the electrospun nanofiber mat on the entire surface of the hydrogel collector, we flattened the outer part of the 3D ear cartilage-shaped hydrogel collector. After fabricating an electrospun nanofiber mat on one side of the hydrogel collector, the 3D ear cartilage-shaped hydrogel collector was flipped to fabricate the electrospun nanofiber mat on the other surface of the 3D ear cartilage-shaped hydrogel collector.

### Characterization of an Electrospun Nanofiber Mat

The nanostructure of an electrospun nanofiber mat on the 3D ear cartilage-shaped hydrogel collector was observed by scanning electron microscopy (SEM; Supra 25, Carl Zeiss, Germany), and diameters of the nanofibers were measured in the SEM image by ImageJ. For the measurement of thickness, an electrospun nanofiber mat with the 3D ear cartilage-shaped hydrogel collector was immersed in the mixture of PDMS monomer and curing agent at a weight ratio of 10:1. And then, the PDMS with the electrospun nanofiber mat was cured in a dry oven at a moderate temperature of 50℃ for 24 h. The PDMS-embedded electrospun nanofiber mat was cross-sectioned, and the thickness of the electrospun nanofiber mat was measured based on the cross-sectional image captured by a microscope (Olympus BX53F2, Olympus, Japan).

### Numerical Simulation

An electric field developed between the metal needle and the collector was numerically simulated by COMSOL Multiphysics v5.0 (COMSOL, USA) software. Three ear cartilage-shaped model collectors made of copper, PLA, and hydrogel were utilized for the numerical simulation. The 3D ear cartilage-shaped collector was simplified as a 2D cross section geometry. A metal ground wire was connected to the 3D ear cartilage-shaped collector. Other geometrical parameters were designated as the actual values of the conformal electrospinning process: (1) distance between the metal needle and the model collector of 20 cm and (2) applied electrical voltage of 19 kV. The hydrogel collector, which was the alginate–gelatin hydrogel, was modeled based on the space charge density of the interstitial fluid of the hydrogel. Mobile ions in the interstitial fluid can be described by the Boltzmann equation, resulting in the space charge density ρ(*x*), as follows [33]:

1 $${\rho }\left({x}\right)=-2e{c}_{0}\,\mathrm{sinh}\left(\frac{e}{{k}_{B}T}{\phi }\left(x\right)\right),$$

where $e$ is the electron charge, ${c}_{0}$ is the electrolyte concentration, ${k}_{B}$ is the Boltzmann’s constant, $T$ is the temperature, and $\phi$ is the electrical voltage. The dielectric constant of the hydrogel collector was set as 70 [34]. To plot the direction of the electric field, a reference line $l\left(\mathrm{x}\right)$ of 10 mm was drawn 10 mm above the recessed region of the 3D ear cartilage-shaped collector.

### Statistical Analysis

Statistical analyses were conducted by the one-way ANOVA analysis using MINITAB v17.1.0 software (MINITAB. LCC, USA). The statistically significance was considered if $p$-value is less than 0.05.

## Results and Discussion

### Hydrogel-Assisted Electrospinning

**Fig. 1:**
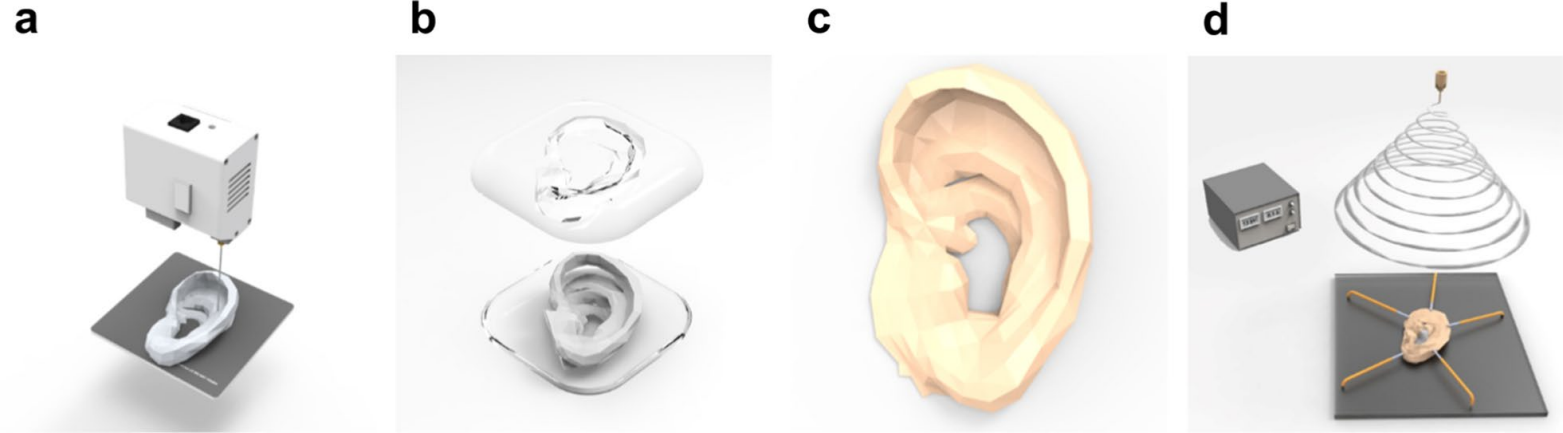
Schematized process of conformal fabrication of an electrospun nanofiber mat on a 3D ear cartilage-shaped hydrogel collector. **a** The 3D printing process for the 3D ear cartilage-shaped template. **b** The PDMS negative mold replicated by the 3D ear-cartilage-shaped template. **c** The alginate–gelatin hydrogel collector with the shape of the ear cartilage. **d** Conformal fabrication of an electrospun nanofiber mat with the flattened 3D ear cartilage-shaped hydrogel collector

Figure 1 schematizes the conformal fabrication of an electrospun nanofiber mat on the 3D ear cartilage-shaped hydrogel collector. Figure 1a shows that the 3D ear cartilage-shaped template was printed by a fused deposition modeling (FDM) 3D printer. As the conformal fabrication process utilized the 3D printer, the printed structure can be freely designed and easily changed to suit a highly complicated shape, such as ear cartilage. Furthermore, the higher-resolution structures could be obtained by adopting stereolithography (SLA) or digital light processing (DLP) 3D printers, which achieved better resolution by using photopolymerization compared to the FDM 3D printer. Figure 1b shows the PDMS negative mold that replicated the 3D ear cartilage-shaped template made by 3D printing. Figure 1c shows the alginate–gelatin hydrogel collector with the shape of ear cartilage by replicating the PDMS negative mold. Figure 1d shows the conformal fabrication of the electrospun nanofiber mat on the 3D ear cartilage-shaped hydrogel collector. When we placed the 3D ear cartilage-shaped hydrogel collector on a flat substrate, the helix of the 3D ear cartilage-shaped hydrogel collector was not contacted with the flat bottom substrate and apart from the substrate due to the complex geometries of ear cartilage, which induced height difference among helix, scapha, and antihelix of the 3D ear cartilage-shaped hydrogel collector. Generally, the height difference due to the protruded part of a 3D electroconductive collector prevented a conformal fabrication of an electrospun nanofiber mat on the 3D electroconductive collector. This is because the protruded part of the 3D electroconductive collector attracts most of the electrospun nanofibers and hamper the deposition of the nanofibers in the lower part of the 3D electroconductive collector [35, 36]. To reduce the influence of the complex geometries of the 3D ear cartilage-shaped hydrogel collector, we flattened the protruded part of the 3D ear cartilage-shaped hydrogel collector to the flat bottom substrate by exploiting the flexibility of the hydrogel. After that, by performing electrospinning on the 3D ear cartilage-shaped hydrogel collector, the electrospun nanofiber mat was deposited conformally on the entire surface of the 3D ear cartilage-shaped hydrogel collector. Finally, the flattened 3D ear cartilage-shaped hydrogel with the electrospun nanofiber mat was returned to its original shape of the ear cartilage. This restoration to the original shape could be possible because the flattening of the hydrogel collector was performed in the elastic deformation region and the nanofiber mat has a negligible influence on the mechanical property of the hydrogel collector with the nanofiber mat due to its lower thickness compared to that of the hydrogel collector.

### Fabrication and Mechanical Property Evaluation of the Alginate–Gelatin Hydrogel

**Fig. 2:**
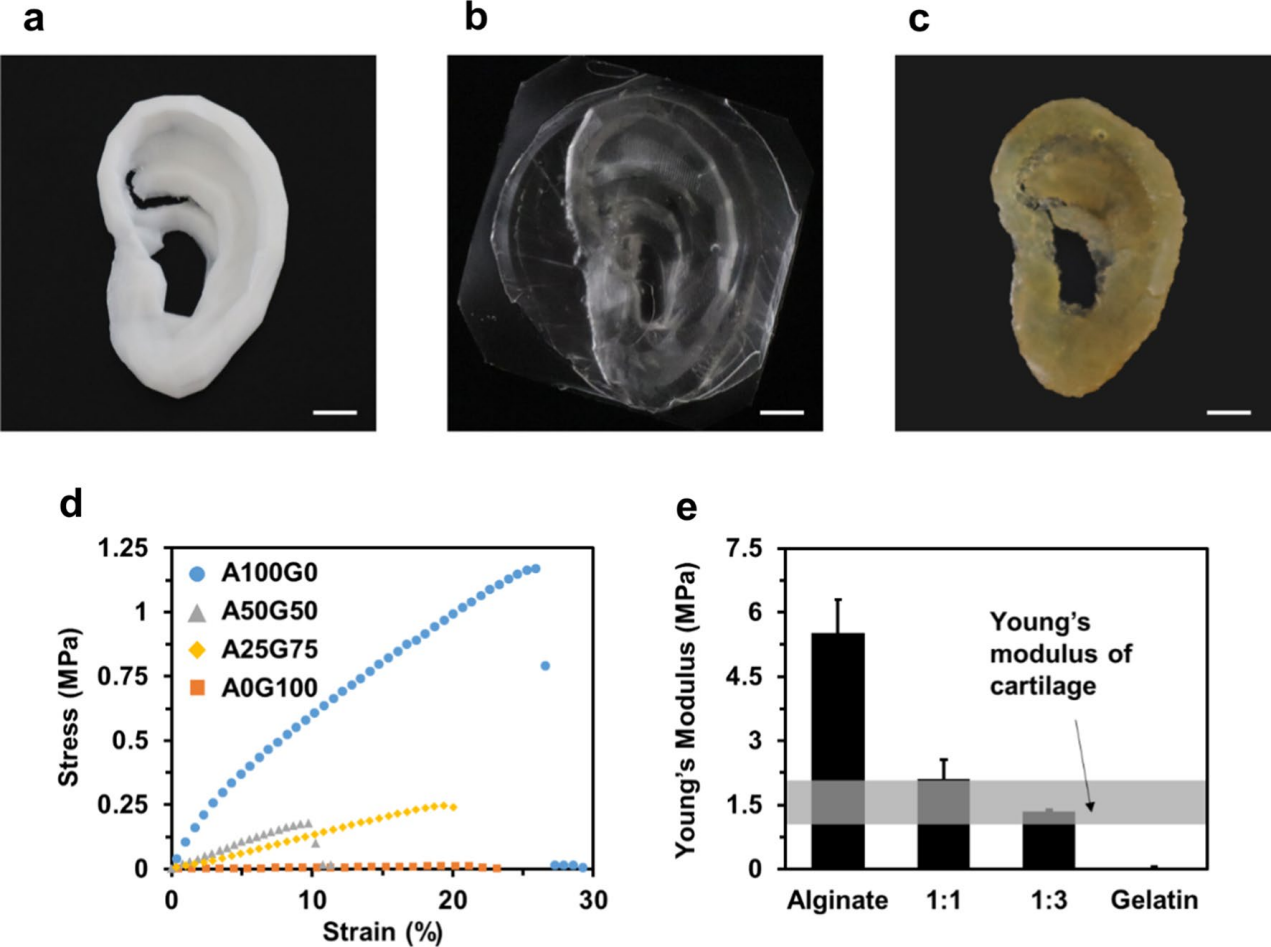
**a** The 3D ear cartilage-shaped template printed by an FDM 3D printer. **b** The PDMS negative mold for the production of the 3D ear cartilage-shaped hydrogel collector. **c** 3D ear cartilage-shaped hydrogel collector made of a mixture of alginate and gelatin. **d** Strain–stress curve of the 4 specimens with the different mixing ratios of alginate and gelatin. **e** Young's modulus of the 4 specimens and the range of Young’s modulus of native ear cartilage (gray region). All scale bars are 1 cm

Figure 2a shows the 3D ear cartilage-shaped template printed with PLA filaments. PLA has sufficient mechanical properties and a high melting temperature (~ 130℃), suitable for the PDMS molding with maintaining the original ear shape. The PDMS negative mold is shown in Fig. 2b. The PDMS was selected as a mold material due to its flexibility required for the demolding of complex geometries of the hydrogel collector. The alginate–gelatin hydrogel collector in Fig. 2c shows the complex structure of the ear, such as helix, scapha, and antihelix. To reveal the broad selection of the mechanical properties of the alginate–gelatin hydrogel collector, we prepared 4 specimens for the mechanical test with the different mixing ratios of alginate and gelatin. Figure 2d, e shows the stress–strain curve and Young’s modulus, respectively, according to the ratio of the hydrogel materials. Figure 2d shows that the specimen made of pure gelatin showed the lowest mechanical strength, and by increasing the content of the alginate, the mechanical strength of the alginate–gelatin mixture was linearly increased. In Fig. 2e, Young’s modulus of alginate–gelatin hydrogel varied from 0.04 MPa to 5.53 MPa. For the case of pure gelatin, named A0G100, the specimen exhibited the lowest Young’s modulus of 0.04 ± 0.01 MPa and thus was difficult to retain its shape during electrospinning. Conversely, the specimen of pure alginate, named A100G0, had the highest Young’s modulus of 5.53 ± 0.77 MPa, capable of maintaining its shape during electrospinning. The A50G50 and A25G75 specimens, which are the mixture of alginate and gelatin, exhibited Young’s modulus of 2.10 ± 0.45 MPa and 1.35 ± 0.03 MPa, respectively. In particular, considering the ear cartilage as a target, Young's modulus of the A25G75 specimen was within Young's modulus range (1–2 MPa) of the native ear cartilage, which is shown as the gray region in Fig. 2e. Based on these results, the ratio of A25G75 was utilized for the 3D ear cartilage-shaped hydrogel collector.

### Fabrication of the Nanofiber Mat on an Alginate–Gelatin Hydrogel Collector

**Fig. 3:**
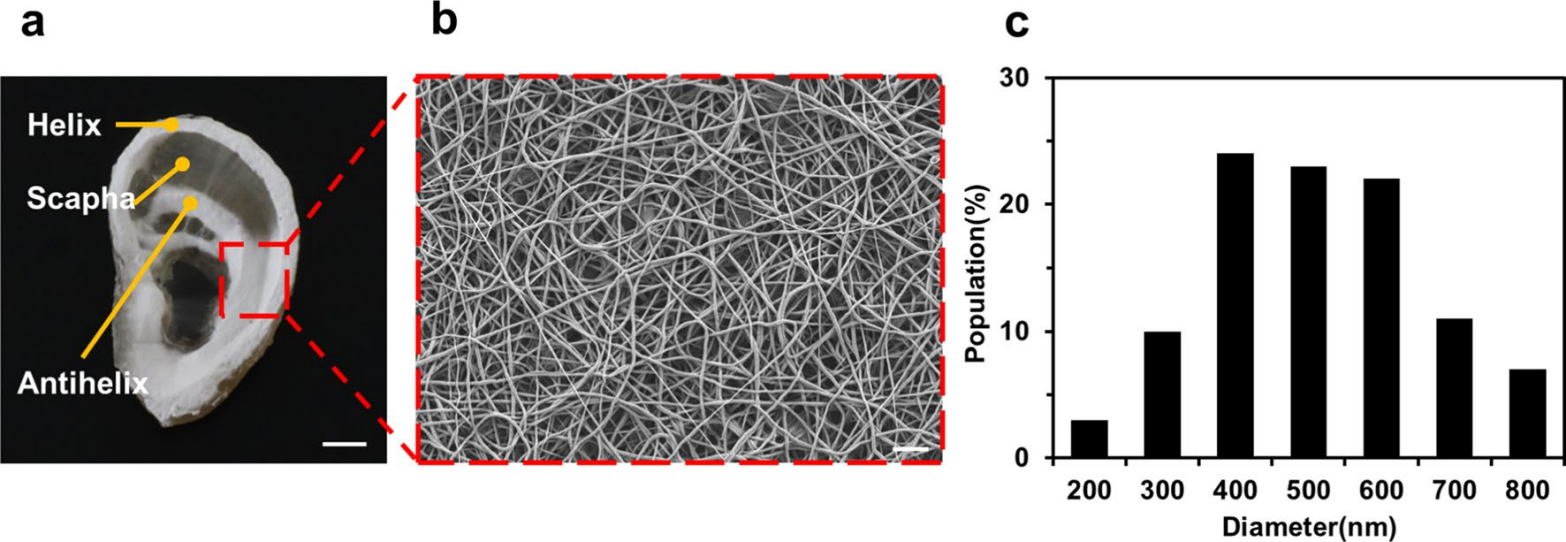
**a** An electrospun nanofiber mat on the 3D ear cartilage-shaped hydrogel collector. **b** A magnified image of the electrospun nanofiber mat on the 3D ear cartilage-shaped hydrogel collector dashed in (**a**). **c** The population of the electrospun nanofibers on the 3D ear cartilage-shaped hydrogel collector. The scale bars are 1 cm (**a**) and 1 µm (**b**)

To investigate the deposition behavior of electrospun nanofibers on the alginate–gelatin hydrogel collector, we performed a numerical simulation of electric field with three types of collectors: a copper collector, a PLA collector, and an alginate–gelatin hydrogel collector. The deposition of electrospun nanofibers was generally determined by the interaction between the charge of the electrospun nanofibers and the electric field. In this sense, the simulation of the electric field has been utilized to understand the deposition of electrospun nanofibers on the collector. In the simulation result with the PLA collector, as shown in Figure S1b, since most of the electric field was concentrated towards the ground wire, it was expected that electrospun nanofibers would not be deposited on the surface of the hydrogel collector. In contrast, in the simulation result with the alginate–gelatin hydrogel collector shown in Figure S1c, the electric field was focused throughout the surface of the alginate–gelatin hydrogel collector, like a copper collector (Additional file 1: Figure S1a). From this simulation result, we expected that electrospun nanofibers would be majorly deposited on the surface of the alginate–gelatin hydrogel collector. This is because the hydrogel collector has a sufficient electrical conductivity due to the mobile ions in the hydrogel and generated a uniform electric field toward the collector, like a metal collector. However, the PLA collector, which is a dielectric material, could not sufficiently attract electric field, and thus, the electric field attracted toward the ground wire, not the PLA collector. These simulation results were confirmed by electrospinning on the PLA and alginate–gelatin hydrogel collector and comparing the thicknesses of an electrospun nanofiber mat (Additional file 1: Figure S1d). Similar to the simulation results with the PLA collector, most of the electrospun nanofibers were placed on a ground wire and the helix part of the PLA collector. At the locations excluding the ground wire and the helix part, electrospun nanofibers on the surface of the PLA collector were stacked in micrometer scale or less. Contrary, the thicknesses of an electrospun nanofiber mat deposited on the helix were measured for the PLA and alginate–gelatin hydrogel collector to compare the nanofiber deposition according to the type of the collector, and the thicknesses of the nanofiber mat on each collector were 3.09 ± 0.37 μm and 33.24 ± 2.43 μm, respectively (Additional file 1: Figure S1d). In the case of the PLA collector shown in Additional file 1: Figure S1b, an electric field was mainly focused on the ground, and electrospun nanofibers were deposited more than 10 times thinner on the PLA collector than the hydrogel collector for the same electrospinning time. Given that PLA had a much lower dielectric constant compared to hydrogel at room temperature, the electric field could not be mainly concentrated on the collector itself, and thus, nanofibers were deposited elsewhere, such as the ground. From this result and our previous study, it is confirmed that the hydrogel collector could sufficiently collect the electric field, and thus, nanofiber mats were deposited thicker on the hydrogel collector compared to the PLA collector. This result implies that the alginate–gelatin hydrogel is an effective collector for electrospun nanofibers during electrospinning. Next, we confirmed that the alginate–gelatin hydrogel collector could produce nanoscale fibers during electrospinning. The electrospun nanofiber mat on the alginate–hydrogel collector is shown in Fig. 3a with the incomplete cover of electrospun nanofibers on the surface of the collector. Figure 3b shows a magnified SEM image of electrospun nanofibers on the alginate–gelatin hydrogel collector. From the SEM image, a high aspect ratio was confirmed with nanoscale nanofiber thickness and micro-scale nanofiber length. Also, defects such as bubbles that can lead to errors when measuring nanofiber thickness were not found, and there was no significant difference in the thickness variation. From these results, we believed that electrospinning with the aforementioned condition was carried out continuously. The average diameter of the fabricated electrospun nanofibers on the alginate–gelatin hydrogel collector was 564 ± 153 nm, and most of the nanofibers have diameters ranging from 400 to 600 nm in Fig. 3c. From the SEM image, electrospun nanofibers showed a high aspect ratio with a nanoscale diameter and a micro-scale length. Thus, this electrospinning technique could be considered as a continuous fiber preparation process.

As shown in Fig. 3a, we observed that the electrospun nanofiber mat has not fully covered the 3D ear cartilage-shaped hydrogel collector, particularly on the scapha, the recessed region between the helix and antihelix. This result indicated that there were void spaces at the electrospun nanofiber mat. Also, electrospun nanofibers were not deposited conformally on the entire surface but suspended as an aligned configuration, which was observed in the inclined gap method [28]. Not only for the shape of the ear cartilage, the complex geometries of the 3D electroconductive collector, such as protrusion or high curvature, would generally cause incomplete covering of an electrospun nanofiber mat on the surface of the collector.

### Numerical Simulation of Conformal Fabrication of an Electrospun Nanofiber Mat on the 3D Ear Cartilage-Shaped Hydrogel Collector

**Fig. 4:**
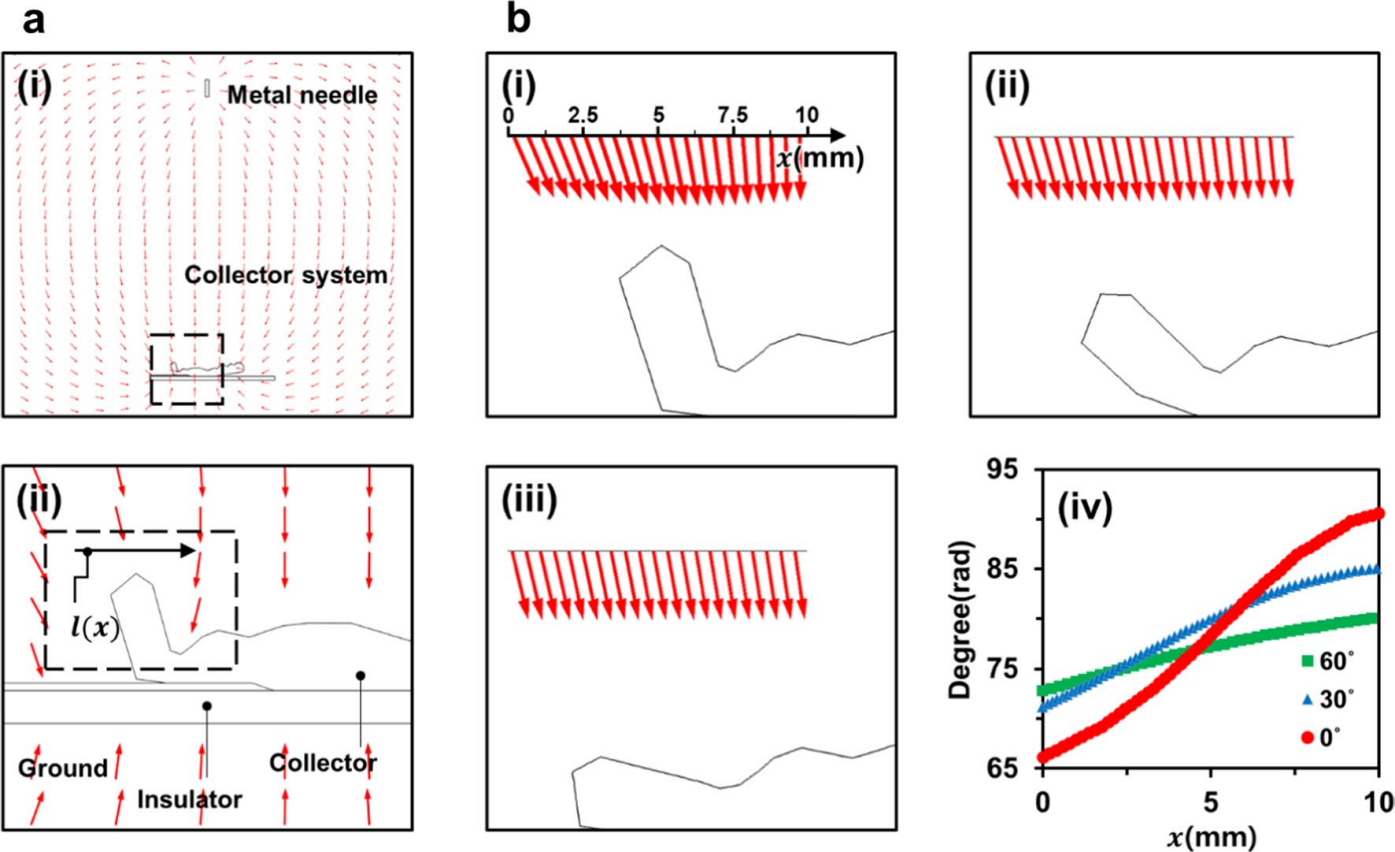
Electric field simulations for the hydrogel collector (**a**-(i)) and a magnified image of the dashed rectangular in **a**-(i) (**a**-(ii)). (**b**) The dashed rectangular in **a**-(ii) showing the recessed region of the hydrogel collector. The electric field arrows along the reference line $l(\mathrm{x})$ with the bending angles of 0° (**b**-(i)), 30° (**b**-(ii)), and 60° (**b**-(iii)). **b**-(iv) The angle of the electric field to the 3D ear cartilage-shaped hydrogel collector with the bending angle of 0°, 30°, and 60° along the reference line $l(\mathrm{x})$

To achieve the conformal fabrication of an electrospun nanofiber mat on the 3D electroconductive collector, we utilized the flexibility of the hydrogel collector, which would not be generally attained by a metal collector. The flexibility of the hydrogel collector enabled it to alter the shape of the collector, thereby reducing the height difference and flattening the hydrogel collector. Firstly, we numerically confirmed the conformal fabrication of an electrospun nanofiber mat on the 3D ear cartilage-shaped hydrogel collector. Figure 4a-(i) shows the configuration of the electrospinning process with the 3D ear cartilage-shaped hydrogel collector. Considering that electrospun nanofibers were difficult to be deposited on the recessed region of the 3D ear cartilage-shaped hydrogel collector, we highlighted the recessed region between the helix and antihelix with a 2D cross section of the 3D ear cartilage-shaped hydrogel collector as shown in Fig. 4a-(ii). The helix was inclined at an angle around 60° to the bottom substrate, thereby forming the recessed region between the helix and antihelix. To alleviate such a recessed region, we reduced the angle by bending the helix of the 3D ear cartilage-shaped hydrogel collector by exploiting the flexibility of the hydrogel collector, unlike a metal collector. The numerical simulation results with the different bending angles of 0°, 30°, and 60° are shown in Fig. 4b-(i), b-(ii), and b-(iii), respectively. Figure 4b-(iv) shows the angle of the electric field along the imaginary line for three cases. The average values in the angle of the electric field with imaginary lines were 79.56°, 79.39°, and 77.26° with the bending angles of 0°, 30°, and 60°, respectively, showing a biased angle with no significant variation between each case. Such a biased angle was caused because the recessed region between the helix and antihelix was the left part of the 3D ear cartilage-shaped hydrogel collector, as shown in Fig. 4a-(i). For the case of the angle deviation of the electric field, the case of the bending angle of 0° showed a deviation of 8.23° along the reference line $l(\mathrm{x})$. In contrast, by bending the helix at 60°, the angle deviation of the electric field was greatly reduced by 2.36°, which was more than 70% alleviated from the angle deviation of the electric field caused by bending 0°. Such large-angle deviation for the case of the bending angle of 0° would be attributed from the focused electric field toward the protruded helix, which resulted in the concentrated deposition of electrospun nanofibers on the helix and thereby hampered the conformal fabrication of an electrospun nanofiber mat on the 3D ear cartilage-shaped hydrogel collector. The reduction of angle deviation by bending the helix relieved such concentrated electric field, and therefore, bending the helix is expected to enable conformal deposition of electrospun nanofibers on the 3D ear cartilage-shaped hydrogel collector.

### Conformal Fabrication of an Electrospun Nanofiber Mat on the 3D Ear Cartilage-Shaped Hydrogel Collector

**Fig. 5:**
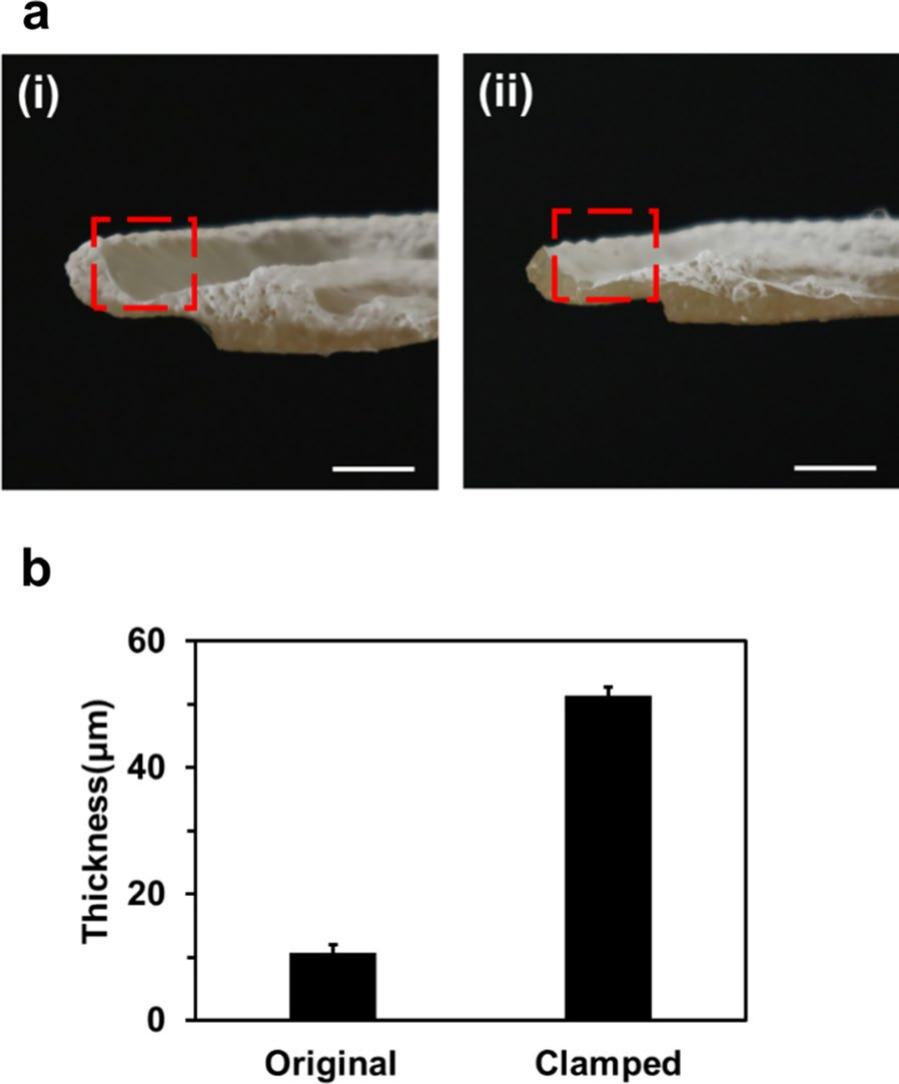
**a** Electrospun nanofiber mats on the scapha of the original hydrogel collector (**a**-(i)) and the flattened hydrogel collector (**a**-(ii)). **b** The thicknesses of the electrospun nanofiber mats at the scapha on the original and the flattened hydrogel collector. All scale bars are 1 cm

To achieve the conformal fabrication of an electrospun nanofiber mat on the 3D ear cartilage-shaped hydrogel collector, the hydrogel collector should be flattened following the simulation result. In this study, we flattened the 3D ear cartilage-shaped hydrogel collector to bend the helix. The helix and outer parts were flattened with metal fixtures, as shown in Figure S2b. The nanofiber-coated hydrogel collector can be returned to its original shape when deformed in the elastic deformation region of the hydrogel. This is because the nanofiber mat has a negligible influence on the mechanical property of the fiber-coated hydrogel collector due to its lower thickness compared to that of the hydrogel collector. The hydrogel collector was deformed in the elastic deformation region, and thus, the nanofiber-coated hydrogel could be restored its original shape. After that, electrospinning was performed on the original and flattened 3D ear cartilage-shaped hydrogel collector. The cross section images of the original and flattened 3D ear cartilage-shaped hydrogel collector after electrospinning are shown in Fig. 5a-(i), (ii).

In the case of the original 3D ear cartilage-shaped hydrogel collector, electrospun nanofibers were suspended between helix and antihelix, not deposited on the scapha of the 3D ear cartilage-shaped hydrogel collector. When the 3D ear cartilage-shaped hydrogel collector was flattened, an electrospun nanofiber mat entirely covered the helix, scapha, and antihelix. Especially, electrospun nanofibers could be deposited on the recessed region between helix and antihelix, which was not possible without flattening the 3D ear cartilage-shaped hydrogel collector. The dramatic thickness difference between the electrospun nanofiber mat on the original and the flattened 3D ear cartilage-shaped hydrogel collector is shown in Fig. 5b. The lower thickness of the electrospun nanofiber mat on the original hydrogel collector showed the retarded deposition on the recessed region, while the flattened hydrogel collector could attract sufficient amount of electrospun nanofibers on the recessed region. With this result, electrospun nanofibers were conformally deposited on the 3D ear cartilage-shaped hydrogel collector even with complex geometries, such as helix and antihelix by flattening the collector.

**Fig. 6:**
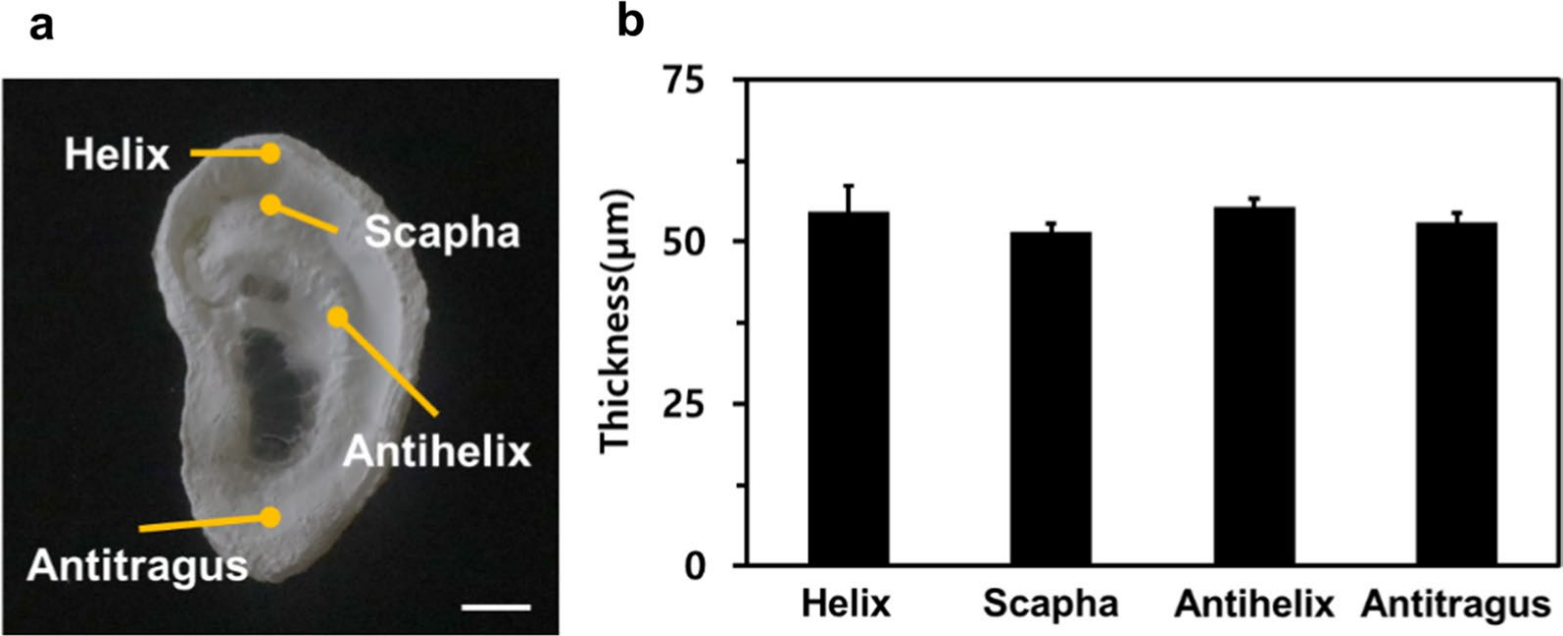
**a** Conformally fabricated nanofiber mat on the 3D ear cartilage-shaped hydrogel collector. The scale bar is 1 cm. **b** The thicknesses of the electrospun nanofiber mat at several positions, including helix, scapha, antihelix, and antitragus on the flattened 3D ear cartilage-shaped hydrogel collector

Lastly, we confirmed the uniformity of an electrospun nanofiber mat on the flattened 3D ear cartilage-shaped hydrogel collector (Fig. 6). Figure 6a shows that an electrospun nanofiber mat could entirely cover the 3D ear cartilage-shaped hydrogel collector without showing any voids for the case of the original 3D ear cartilage-shaped hydrogel collector. Figure 6b shows the thickness of the electrospun nanofiber mat deposited at the helix (54.58 ± 3.99 μm), the antihelix (55.40 ± 1.17 μm), the antitragus (53.05 ± 1.39 μm), and the scapha (51.49 ± 1.24 μm), where the nanofibers were not deposited with the original 3D ear cartilage-shaped hydrogel collector. As a result of the electrospinning with a flattened 3D ear cartilage-shaped hydrogel collector, we could confirm that the electrospun nanofiber mat was deposited conformally and uniformly on the hydrogel collector. Furthermore, based on the results of the previous hydrogel-assisted electrospinning study, we are convinced that the thickness of the fabricated 3D conformal nanofiber mats could be controlled by exploiting a hydrogel collector. As a future perspective, given that the 3D ear cartilage-shaped hydrogel with this nanofiber mat possessed mechanical properties similar to those of native ear cartilage and has a biomimetic nanostructure, it could be expected to be applied as an artificial ear cartilage implant. Considering the utilization to tissue engineering, the residual charge in the nanofiber mat should not be neglected which might influence the cell behaviors. We believed that this conformal fabrication of an electrospun nanofiber mat is pioneering work to produce a 3D nanofiber membrane, and thus, could be utilized in a broad range of applications suggesting a novel type of nanofiber assemblies such as 3D native-tissue mimicking scaffold and 3D porous membrane for efficient filtering.

## Conclusions

In summary, we developed the conformal fabrication of an electrospun nanofiber mat on the 3D ear cartilage-shaped hydrogel collector that has the recessed region. As a result, the nanofiber mat on the 3D ear cartilage-shaped hydrogel collector was produced with the fully replicated shape of the collector. Most importantly, the utilization of the flexibility of the hydrogel collector allowed to adjust the recessed region of the collector, and thereby, an electrospun nanofiber mat was conformally deposited with the uniform thickness on the entire surface of the collector which would not be covered with the conventional electrospinning. Given that this conformal fabrication technique would be compatible with a variety of hydrogel materials, this technique could be a more versatile and effective technique for fabricating conformal nanofiber mat in the various fields of tissue engineering, drug/cell delivery, clothing, and battery.

## Supplementary Information

**Additional file 1:** The following files are available free of charge. Numerical simulation of electric fields with three types of collectors; and fabricated 3D ear cartilage-shaped alginate-gelatin hydrogel collector (PDF).

## Data Availability

All data supporting the conclusions of this article are included within the article and supplementary document.

## Abbreviations

3D: Three-dimensional
PDMS: Polydimethylsiloxane
PLA: Polylactic acid
PCL: Polycaprolactone
PMMA: Polymethyl methacrylate
SEM: Scanning electron microscopy
FDM: Fused deposition modeling
SLA: Stereolithography
DLP: Digital light processing
A100G0: Pure alginate
A0G100: Pure gelatin
A50G50: Alginate/gelatin = 50:50
A25G75: Alginate/gelatin = 25:75
