# Supplementary material for: Conformal Fabrication of an Electrospun Nanofiber Mat on a 3D Ear Cartilage-Shaped Hydrogel Collector Based on Hydrogel-Assisted Electrospinning
Source: Nanoscale Res Lett. 2021 Jul 9;16:116. doi: 10.1186/s11671-021-03571-6 (PMC8271053; doi:10.1186/s11671-021-03571-6)
Supplement: Supplementary file 1 — Additional file 1: The following files are available free of charge. Numerical simulation of electric fields with three types of collectors; and fabricated 3D ear cartilage-shaped alginate-gelatin hydrogel collector (PDF). [file 11671_2021_3571_MOESM1_ESM.docx]

Supplementary Information for

**Conformal fabrication of an electrospun nanofiber mat on a 3D ear cartilage-shaped hydrogel collector based on hydrogel-assisted electrospinning**

*Jin Yeong Song^1^, Hyun Il Ryu^1^, Jeong Myeong Lee^1^, Seong Hwan Bae^2,3^, Jae Woo Lee^2^, Changryul Claud Yi^2,3,**^, Sang Min Park^1,*^*

^1^School of Mechanical Engineering, Pusan National University, 2, Busandaehak-ro 63 beon-gil, Geumjeong-gu, Busan, 46241, South Korea

^2^Department of Plastic and Reconstructive Surgery, Pusan National University School of Medicine, 179 Gudeok-ro, Seo-gu, Busan 49241, South Korea

^3^Biomedical Research Institute, Pusan National University Hospital, 179 Gudeok-ro, Seo-gu, Busan 49241, South Korea

Corresponding Author

* Prof. Sang Min Park

e-mail: sangmin.park@pusan.ac.kr

** Prof. Changryul Claud Yi

e-mail: fuffl0000@gmail.com

**1. Numerical simulation of electric fields with three types of collectors**


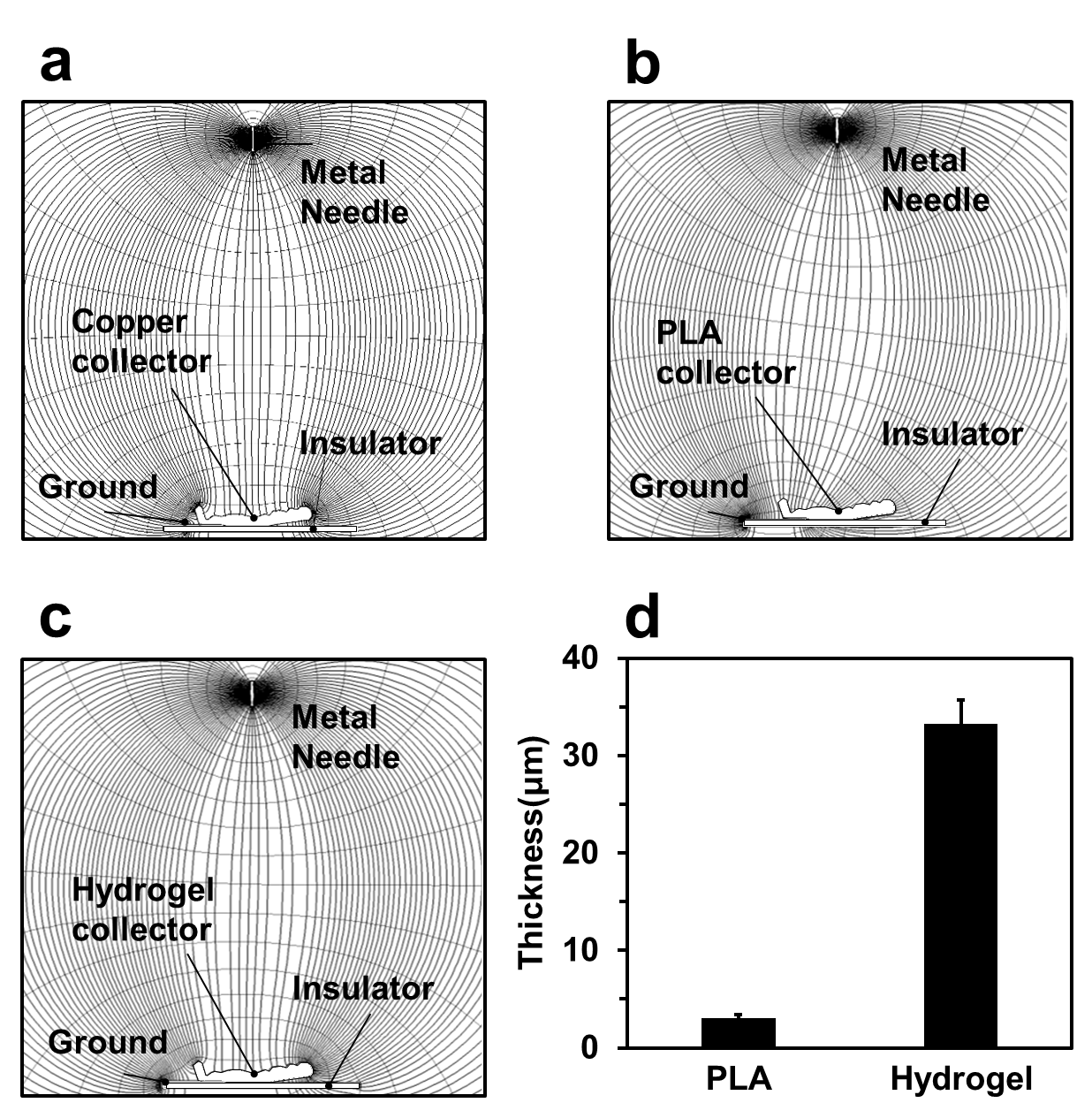


**Figure S1.** Simulation result of the electric field with a copper collector (a), a PLA collector (b), and an alginate-gelatin hydrogel collector (c). (d) The thicknesses of the electrospun nanofiber mat on the PLA collector and the alginate-gelatin hydrogel collector.

**2. Fabricated 3D ear cartilage-shaped alginate-gelatin hydrogel collector**


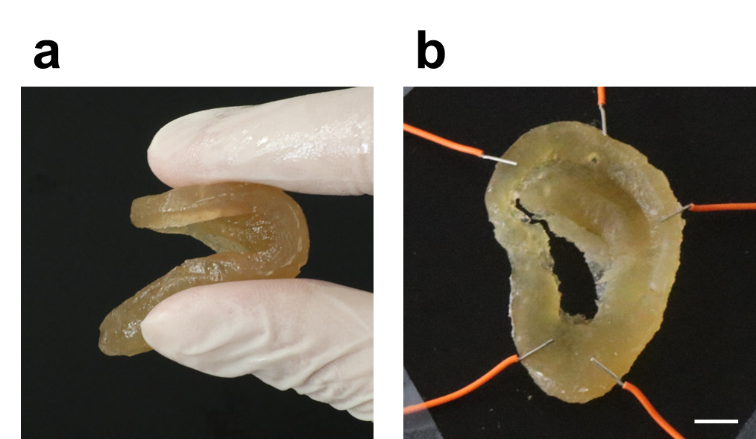


**Figure S2.** (a) Manual deformation of the 3D ear cartilage-shaped hydrogel collector. (b) The flattened 3D ear cartilage-shaped collector with metal fixtures. The scale bar is 1 cm.
